# Construction of a high-density linkage map and mapping of sex determination and growth-related loci in the mandarin fish (*Siniperca chuatsi*)

**DOI:** 10.1186/s12864-017-3830-3

**Published:** 2017-06-06

**Authors:** Chengfei Sun, Yongchaox Niu, Xing Ye, Junjian Dong, Wushu Hu, Qingkai Zeng, Zhihang Chen, Yuanyuan Tian, Jin Zhang, Maixin Lu

**Affiliations:** 1 Key Laboratory of Tropical & Subtropical Fisheries Resource Application & Cultivation, Ministry of Agriculture, Pearl River Fisheries Research Institute, Chinese Academy of Fishery Sciences, No. 1, XingYu Road, Xi Lang, Fang Cun, PO, Guangzhou, Guangdong 510380 China; 2 Total Genomics Solution (TGS) Institute, Shengzhen, Guangdong, 518000 China; 3 College of Fisheries and Life Science, Shanghai Ocean University, Shanghai, 201306 China; 4 YuShun Fisheries Company, Qingyuan, Guangdong 511500 China

**Keywords:** *Siniperca chuatsi*, Linkage map, Sex determination, Growth-related trait, Quantitative trait locus

## Abstract

**Background:**

The mandarin fish (*Siniperca chuatsi*) is an important and widely cultured fish in China. However, the lack of selective breeding of mandarin fish in previous decades has resulted in a decline in the growth rate of pond-cultured fish, a shortened period of sexual maturity, and reduced disease resistance; these issues seriously affect the quality and safety of the fish products. Therefore, it is necessary to establish a selective breeding program for the mandarin fish to improve the economical traits of the fish and to sustain the development of the mandarin fish industry.

**Results:**

We constructed a high-density linkage map for it based on double digest restriction site associated DNA sequencing (ddRAD-Sequencing). This map contained 3283 dimorphic single nucleotide polymorphism markers and 24 linkage groups (LGs). The total map-length was 1972.01 cM, with an average interlocus distance of 0.61 cM. One significant quantitative trait locus (QTL) for sex determination trait was detected on LG23, which was supported by five markers, clustered between 60.27 and 68.71 cM. The highest logarithm of odds value (17.73) was located at 60.27 cM, near the marker r1_73194, accounting for 53.3% of the phenotypic variance. Genotypes of all the male fish on r1_33008 were homozygous, whereas those of all females were heterozygous. Thus, LG23 was considered a sex-related linkage group. Eleven significant QTLs, for three growth traits, at two growth stages and the increased values were distributed on four LGs; their contributions to the phenotypic variation were quite low (12.4–17.2%), suggesting that multiple genes affected the growth traits.

**Conclusion:**

This high-resolution genetic map provides a valuable resource for fine-mapping of important traits and for identification of sex-related markers that should facilitate breeding of all-female mandarin fish for aquaculture and mechanistic studies on sex determination.

**Electronic supplementary material:**

The online version of this article (doi:10.1186/s12864-017-3830-3) contains supplementary material, which is available to authorized users.

## Background

The mandarin fish *Siniperca chuatsi* (Basilewsky) (order Perciformes, family Serranidae, subfamily Sinipercinae) is specifically distributed in rivers of some East Asian countries, including China, Vietnam, and Korea [1]. It is a popular food in East Asia and has a long history; its use as a foodstuff is recorded in many ancient Chinese poems and books [2]. Artificial propagation of mandarin fish has been carried out since the 1970s in China and has considerably promoted the development of culture techniques for this fish. Presently, the mandarin fish has become a valuable species, widely cultured in China, with an annual production of approximately 300,000 tons [3]. However, as with many other cultured fish species, the lack of selective breeding of mandarin fish in previous decades has resulted in a decline in the growth rate of pond-cultured fish, a shortened period of sexual maturity, and reduced disease resistance; these issues seriously affect the quality and safety of the fish products [4]. Therefore, it is necessary to establish a selective breeding program for the mandarin fish to improve the economical traits of the fish and to sustain the development of the mandarin fish industry.

Teleosts exhibit a considerably wide range of sex-determination patterns, which may be influenced by the environmental parameters or may be determined genetically. Various genetic mechanisms for sex determination are employed in fish, such as male (the XX-XY system) or female heterogamy (the ZW–ZZ system) [5]. Additionally, sex chromosome differentiation in fish occurs in different stages and displays various mechanisms. Some fish sex chromosomes are similar to autosomes, i.e., they carry many functional genes, and are at a very early evolutionary stage of sex determination. Some fish have a Y-chromosome harboring repetitive DNA sequences and many functional genes, showing similar morphology to the autosomes [6]. However, the fish sex chromosome differentiation and sex determination mechanisms remain to be elucidated.

In the majority of fish, the sex chromosomes are usually small in size or are not sufficiently morphologically divergent to be identified by classical karyotyping. Among the 1700 fish species that have been investigated cytogenetically, only 170 (10.4%) have been reported to have obvious sex chromosomes [6]. Chromosomal karyotype analysis showed that the diploid chromosome number of the mandarin fish is 48 (2n = 48). These chromosomes are classified into two groups: submetacentric or subtelocentric chromosomes (12 pairs) and telocentric chromosomes (12 pairs). However, no dimorphism has been observed in the sex chromosomes of the mandarin fish [7].

Fish species often exhibit significant sexual dimorphism. Commercial fish species [8], such as the Atlantic halibut (*Hippoglossus hippoglossus*) [9], turbot (*Scophthalmus maximus* L.) [10], Japanese flounder (*Paralichthys olivaceus*) [11], tilapia (*Oreochromis niloticus* L.) [12, 13], Salmonidae (*Salmo salar*) [14], and half-smooth tongue sole (*Cynoglossus semilaevis*) [15], display significant variations in size and growth rates between female and male individuals, substantially affecting their commercial value. The females of mandarin fish also display better growth performance (by 10–20% for body weight) than the males. Therefore, screening of sex-associated markers will shorten the time required for the development of all-female mandarin fish for aquaculture and will be helpful in elucidating the mechanisms of sex determination.

Linkage mapping is critical for identifying the location of genes related to quantitative traits, such as those involved in disease resistance, growth, and sex determination. The advent of next-generation sequencing (NGS) has revolutionized the genomic approaches to biology. We can now obtain genome-wide genetic markers using several NGS methods, including reduced-representation libraries, restriction-site associated DNA sequencing (RAD-Seq) [16, 17], genotyping by sequencing, and others. Moreover, these methods are applicable to both model and non-model organisms. Using the NGS methods mentioned above, researchers have constructed genetic maps of several fishes, including the Atlantic halibut [7], Asian seabass (*Lates calcarifer*) [18], Nile tilapia [19], Atlantic salmon [20], turbot [10], Japanese flounder [11] and large yellow croaker (*Larimichthys crocea*) [21]. Moreover, quantitative trait loci (QTL) for growth, gender, and disease resistance of these fish species have been detected using the NGS methods. These applications demonstrate that the NGS methods are effective for the construction of high-density genetic linkage maps and for QTL mapping.

It is evident from the literature that approximately half of the RAD-Seq data (reads without a restriction enzyme site in paired-end sequencing) analyzed without a reference genome are discarded [22, 23]. The successful construction of genetic maps using double digest RAD-Seq (ddRAD-Seq) indicates that it is an efficient and low-cost method for de novo SNP discovery and genotyping in non-model species compared to the existing RAD-Seq approaches [24]. In this study, the ddRAD-Seq technique was applied for the construction of a high-density single nucleotide polymorphism (SNP)-based linkage map as well as for fine-mapping of QTLs for sex determination and growth-related traits in the mandarin fish. Our results will facilitate identification of potential genes determining sex and growth traits and selective breeding of mandarin fish, and they will also be useful in the future de novo genome sequence assembly in the mandarin fish and in the comparative genomic studies of perciform fishes.

## Results

### Construction and sequencing of ddRAD libraries

A total of 157 ddRAD libraries were constructed from the two parents and their 155 offspring and sequenced on an Illumina HiSeqXten platform to generate 753.13 million clean reads, comprising approximately 91.86 Gb of sequencing data. The female and male parental data sets contained 13.66 million filtered reads (comprising 1.67 Gb of data with a GC% of 43.33) and 10.82 million filtered reads (comprising 1.33 Gb of data with a GC% of 42.68), respectively. From the 155 offspring, 728.65 million filtered reads (average of 4.70 million) corresponding to 88.86 Gb of data (average of 573. 30 Mb) were produced (ranging from 192.11 to 1373.38 Mb) for SNP detection (Additional file 1: Table S1).

### SNP calling and genotyping

A total of 16,816 raw polymorphic markers were detected using the STACKS pipeline. After stringent selection (See Methods below), 3283 polymorphic markers were successfully genotyped in both parents and offspring. These SNPs were classified into three categories: maternal heterozygous (1052 SNPs), paternal heterozygous (1762 SNPs), and heterozygous in both (469 SNPs). All these SNPs are listed in Additional file 2: Table S2.

### High-resolution genetic map construction

**Fig. 1:**
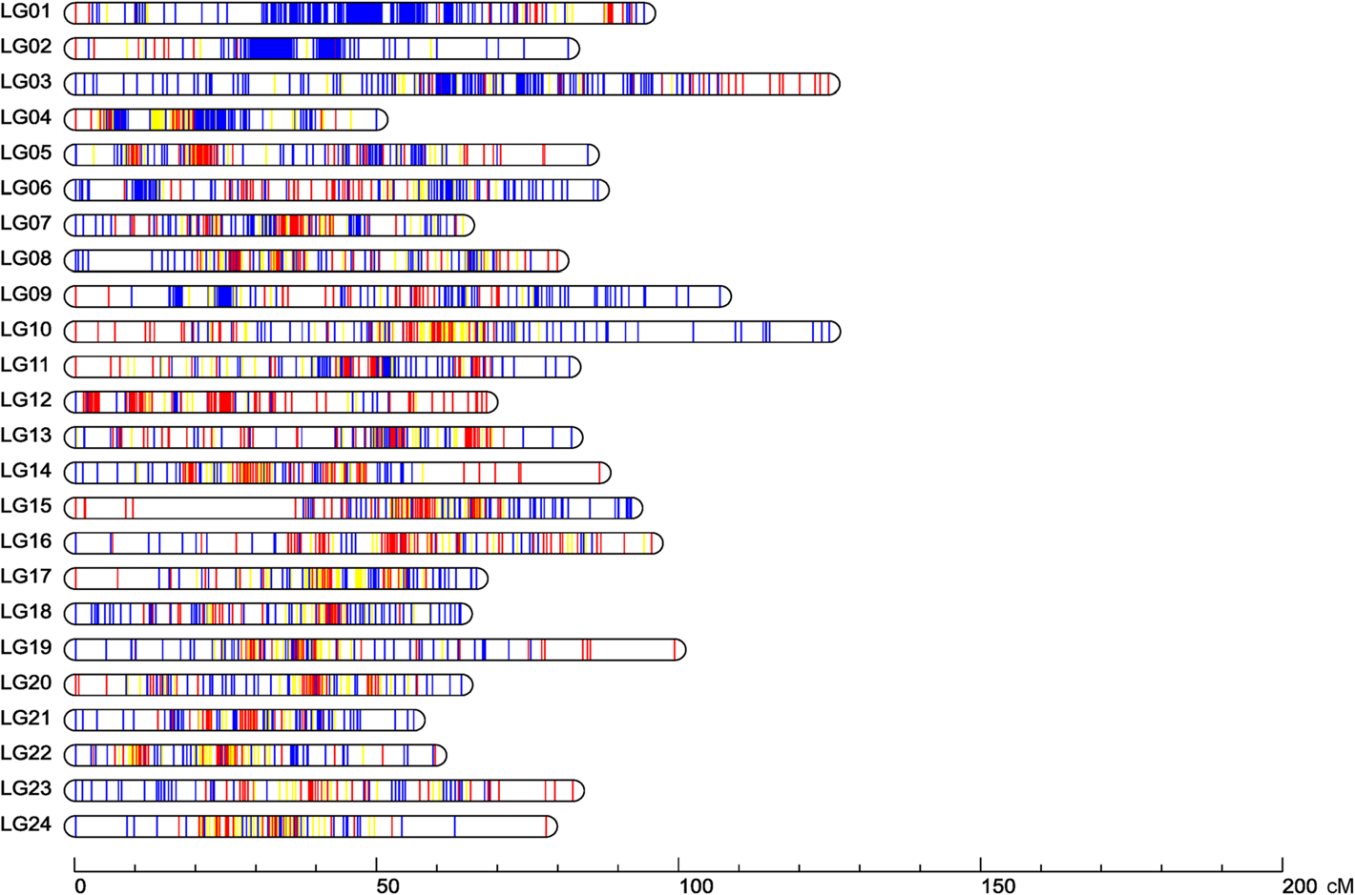
Linkage group lengths and marker distributions of the high-resolution ddRAD-based SNP linkage map of the mandarin fish. Within each linkage group, red, blue, and yellow lines represent maternal heterozygous SNPs, paternal heterozygous SNPs, and SNPs heterozygous in both the parents, respectively. The details of the genetic map are given in Additional file 3: Table S3

**Fig. 2:**
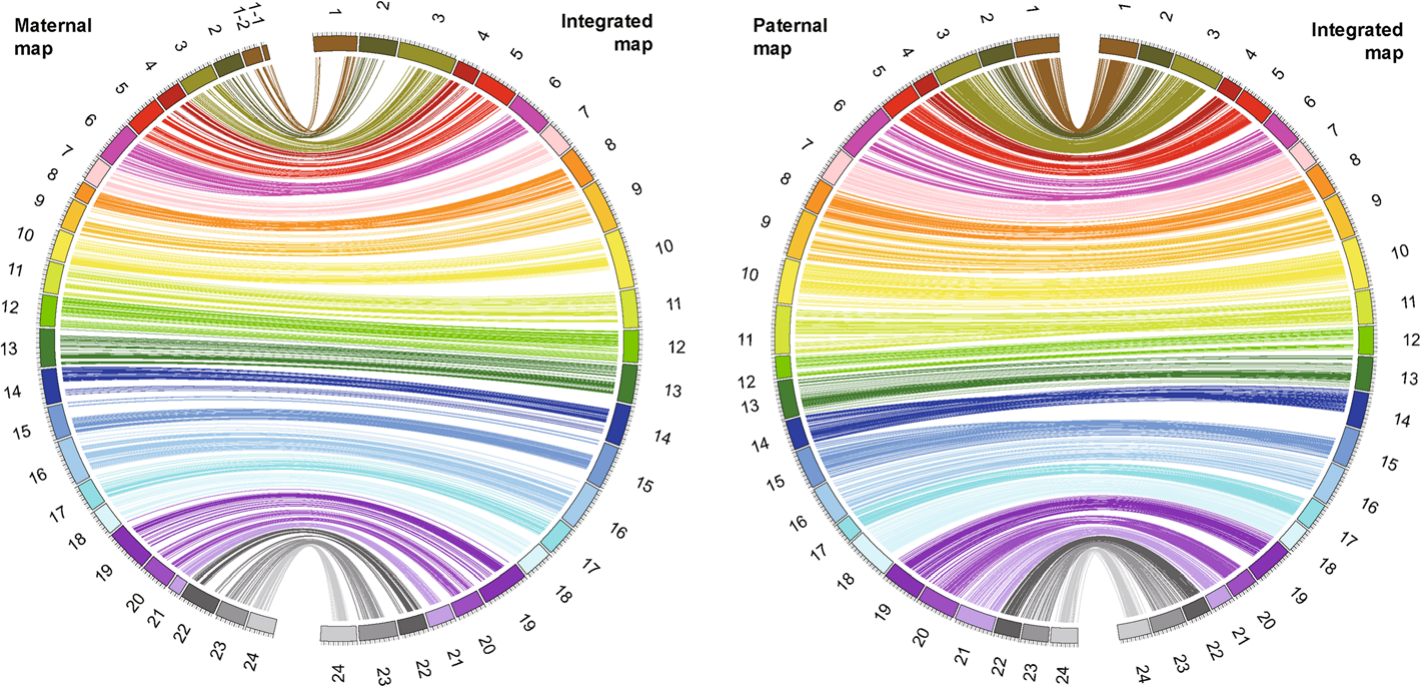
Circos diagram representing the syntenic relationships between the integrated map and the maternal map a or paternal map b of the mandarin fish. Each colored arc represents a marker match between the two maps

A high-resolution ddRAD-based linkage map of the mandarin fish was constructed using a pseudo-testcross strategy (a mapping population is developed by hybridizing two unrelated highly heterozygous parents to produce a set of F1 progeny)^11^. The resulting integrated map consisted of 24 linkage groups (LGs), including 3283 segregating SNPs. The total map length was 1972.01 cM, with an average interlocus distance of 0.61 cM; the genetic length of each LG ranged from 49.84 cM (LG4) to 124.75 cM (LG10), with an average interlocus distance of 0.27–0.93 cM (Table 1 and Fig. 1). The locus names and SNP positions on the 24 LGs of the integrated genetic map are listed in Additional file 3: Table S3. The maternal map contained 1505 SNPs with a total genetic distance of 1576.19 cM. The length of each LG ranged from 10.81 cM (LG1–1) to 97.51 cM (LG16), with an average genetic length of 1.05 cM (Additional file 4: Table S4 and Additional file 5: Fig. S1). The corresponding paternal map consisted of 2215 SNPs representing a total length of 2115.57 cM and ranging from 50.04 cM (LG17) to 131.40 cM (LG6) (Additional file 6: Table S5 and Additional file 5: Fig. S2). A synteny analysis among the integrated map, maternal map, and paternal map was also performed. The order of markers in the three maps was highly consistent (Figure 2) [25].

**Table 1:** Characteristics of the genetic maps of the mandarin fish

LG ID	Integrated map			Maternal map		Paternal map	
	No. of SNPs	Distance (cM)	Average interlocus distance (cM)	No. of SNPs	Distance (cM)	No. of SNPs	Distance (cM)
1	261	94.14	0.36	7 + 22^a^	10.81 + 39.09^a^	242	108.71
2	226	81.55	0.36	17	59.85	214	83.11
3	208	124.64	0.60	47	75.11	178	111.26
4	184	49.84	0.27	64	54.63	156	52.89
5	147	84.81	0.58	77	75.28	84	86.16
6	145	86.47	0.60	49	85.92	108	131.40
7	138	64.17	0.47	76	54.52	80	82.28
8	137	79.77	0.58	79	36.84	81	77.58
9	136	106.68	0.78	37	65.02	106	117.52
10	134	124.75	0.93	77	64.59	89	108.25
11	128	81.77	0.64	65	66.56	77	116.15
12	126	68.04	0.54	104	66.81	31	54.04
13	125	82.11	0.66	80	80.07	63	92.37
14	123	86.75	0.71	78	75.13	66	71.88
15	122	92.02	0.75	70	70.82	70	94.70
16	120	95.38	0.79	89	97.51	44	95.31
17	114	66.41	0.58	68	58.59	81	50.04
18	113	63.81	0.56	55	56.61	73	107.91
19	112	99.17	0.89	65	91.45	68	92.12
20	110	63.90	0.58	68	61.84	65	93.69
21	102	56.00	0.55	46	27.27	68	94.18
22	97	59.58	0.61	63	77.48	58	61.87
23	88	82.36	0.94	48	64.37	54	65.39
24	87	77.89	0.90	54	60.02	59	66.76
Total	3283	1972	0.60	1505	1576.20	2215	2115.57
Average	137	82.17	0.60	63	65.68	92	88.15

^a^LG1 in the maternal map was divided into two LGs due to weak linkage between the distant markers

### Sex determination loci detection

**Fig. 3:**
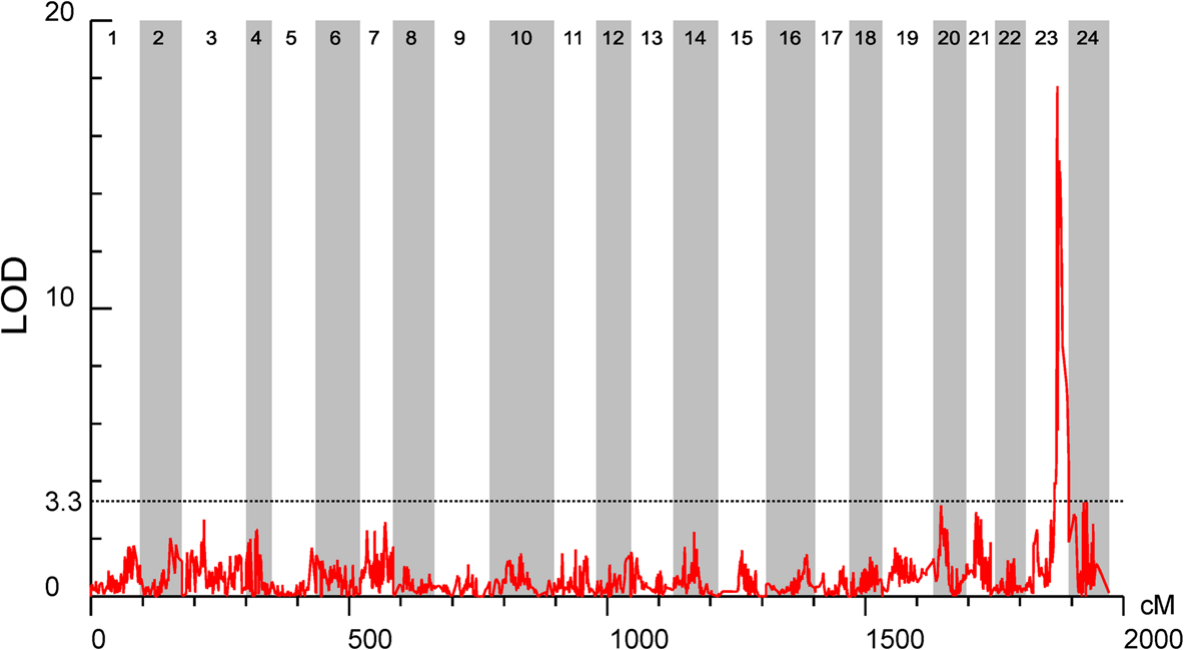
Detection of the sex determination locus in the mandarin fish. LOD significance threshold levels were determined on the basis of 1000 permutations at a significance level of *P* < 0.05

The significant threshold estimated using permutation tests was 3.3 for the sex determining trait (*P* < 0.05). One significant QTL for the sex determining trait was detected on LG23 of the mandarin fish (Table 2 and Figure 3). It was supported by five markers that were clustered together between 60.27 and 68.71 cM on LG23. Among these, the highest LOD value of 17.73 was located at 60.27 cM near the marker r1_73194, which contributed to 53.3% of the phenotypic variation.

**Table 2:** Characteristics of the sex determination QTL in the mandarin fish

Traits	Nr	QTL	LG ID	Genetic position	Peak locus	LOD	Exp%^a^
Gender	4129	Gender-1	23	60.265	r1_73194	17.73	53.3
Gender	4136	Gender-1	23	62.851	r2_237649	13.59	44.5
Gender	4139	Gender-1	23	63.967	r2_42410	15.13	50
Gender	4141	Gender-1	23	65.431	r1_33008	14.76	44.9
Gender	4148	Gender-1	23	68.707	r1_99172	11.58	37.1

^a^Exp%, percentage of the explained phenotypic variation

### Growth-related QTLs

In this study, three growth-related traits, total body length, body weight, and body height were investigated. Each trait was measured at 60 and 195 days (see Methods). The results of the first measurement of total body length, body weight, and body height were designated as FL, FW, and FH and those of the second measurement as SL, SW, and SH, respectively. The increases in total body length, body weight, and body height (the difference of the second and first measurements) were recorded as IL, IW, and IH. Statistical analysis was performed with SPSS software with K-S test (Kolmogorov–Smirnov test). The Asymptotic Significance (2-tailed) was accepted when *P* > 0.05. The results showed that all these traits do not statistically differ from a normal distribution (Additional file 7: Table S6). The estimated significant thresholds for the growth-related traits as determined using the permutation tests were 3.4 (*P* < 0.05).

In total, eleven significant QTLs for three growth-related traits at the first growth stage and the increased values were found to be distributed on four different LGs (Table 3 and Additional file 5: Fig. S3). The highest QTLs for FL, FW, and FH were identical, located at 12.30 cM of LG10, near the marker r1_32378, and correspondingly contributed to 16.9%, 17.2%, and 16.7% of the phenotypic variation, respectively. Marker r1_63885, r1_98745, r1_62529 on LG16 was associated with both IL and IH and contributed to 14.9%, 14.6%, 13.9% and 16.7%, 16.6%, 15.8% of the phenotypic variation, respectively.

**Table 3:** Characteristics of the growth-related QTLs in the mandarin fish (LOD > 3.4)

Traits	Nr	QTL	LG ID	Genetic position	Peak locus	LOD	Exp%^a^
FL	1011	Growth-1	4	28.703	r2_107263	3.68	14.7
FL	1966	Growth-2	10	12.302	r1_32378	3.77	16.9
FL	3146	Growth-3	16	75.207	r1_23321	3.6	13.9
FL	3523	Growth-4	19	35.17	r1_38594	3.41	12.8
FW	1966	Growth-5	10	12.302	r1_32378	3.93	17.2
FH	1966	Growth-6	10	12.302	r1_32378	3.74	16.7
FH	1973	Growth-6	10	17.978	r1_63812	3.45	12.9
IL	3038	Growth-7	16	51.254	r1_75760	3.4	12.4
IL	3083	Growth-7	16	52.079	r1_78507	3.65	13.4
IL	3095	Growth-8	16	53.187	r1_63885	4.15	14.9
IL	3103	Growth-8	16	54.097	r1_98745	3.91	14.6
IL	3107	Growth-8	16	54.756	r1_62529	3.85	13.9
IL	3110	Growth-9	16	56.141	r1_23000	3.51	14.1
IH	3081	Growth-10	16	51.63	r1_66472	3.75	13.7
IH	3095	Growth-11	16	53.187	r1_63885	4.7	16.7
IH	3103	Growth-11	16	54.097	r1_98745	4.42	16.6
IH	3107	Growth-11	16	54.756	r1_62529	4.43	15.8
IH	3109	Growth-11	16	55.655	r1_94016	4.19	14.8

^a^Exp%, percentage of the explained phenotypic variation

Five QTLs for the increased values IL and IH were detected. One major cluster containing three QTLs of IL supported by six markers was detected in the narrow region of 51.25–56.14 cM on LG16. Among them, the highest LOD value of 4.15 was located at 53.19 cM of LG16 near the marker r1_63885, contributing to 14.90% of the phenotypic variation. Interestingly, one QTL of IH supported by a single marker (r1_66472) and the other QTL of IH supported by four markers, were also detected on LG16 in the narrow region of 51.63–55.66 cM, nearly at the same position as the QTL for IL.

In addition, the trends of the QTL curves were very similar for FL, FW, and FH as well as for SL, SW, and SH. The trends of the QTL curves for IL, IW, and IH were similar and corresponded to those of the second measurement (Additional file 5: Fig. S3).

## Discussion

In the present study, a high-resolution genetic linkage map was constructed with 3283 SNPs using the ddRAD-Seq technique. This is the first high-density mandarin fish genetic map reported. The paternal and maternal linkage maps of the mandarin fish were also constructed. The synteny analysis showed that both the paternal and maternal maps had a good synteny with the integrated map, reflecting the high qualities of these three linkage maps. This linkage map enabled us to determine the number and position of the QTLs for growth traits and sex determination, and will provide an important tool for fine-mapping of QTLs in molecular breeding of the mandarin fish.

In this study, the high-resolution genetic map enabled us to successfully detect the sex determination QTLs. One major QTL (LG23) related to sex determination was supported by five markers that were found to cluster at a narrow linkage span (60.27–68.71 cM) of LG23, contributing to 37.1–53.3% of the phenotypic variation, indicating that this was the sex determination QTL in the mandarin fish. Similar phenomena have been reported in some other fish species. In the Atlantic halibut, four markers significantly related to sex determination were found to be located within a 3.2-cM region of LG13, showing 78–89% association with sex (*P* < 0.001) [9]. Additionally, in Gilthead sea bream (*Sparus aurata* L.), two significant QTLs affecting sex determination were found to be located in LG21 [26]. In contrast, for some other fish species, sex-related QTLs were found to be distributed in different LGs. In the turbot, most sex-related QTLs are found in LG21, with a linkage span of 70.882 cM, and a small number of QTLs with high feasibility are also observed in LG7 and LG14 [27]. In the half-smooth tongue sole, seven sex-related loci were mapped in LG1f, LG14f, and LG1m by CIM, accounting for 12.5–25.2% of the trait variation [15]. In the European sea bass (*Dicentrarchus labrax*), putative sex-determining QTLs were found in LG6, LG11, and LG18–21, accounting for 16.19–21.28% of the trait variation [28]. In tilapia species, the sex-determining QTLs were determined to involve at least three chromosomes, i.e., chromosomes 1, 3, and 23 [12, 29–31]. These findings suggest that, in some fish species, multiple LGs or chromosomes are involved in sex determination and provide support to the polygenic sex determination. In the present study, the high clustering of the five markers supporting a major sex-related QTL in the mandarin fish suggested that a single LG may be involved in sex determination and that the sex determination genes may be located in this LG.

Further genotyping of the sex determining QTLs in the present study revealed that the genotypes of all the male fish on locus r1_33008 were homozygous, whereas those of the females were heterozygous. Similar results have also been reported for the Nile tilapia*.* At two loci (Oni23063 and Oni28137), the genotypes of female tilapia were homozygous for the same allele, whereas those of the male individuals were heterozygous [32]. Thus, detection of the sex-specific marker locus r1_33008 provides a useful molecular tool for distinguishing male and female fish and may facilitate the cultivation of all-female mandarin fish.

In this study, a total of 11 QTLs related to three growth traits were discovered. However, compared to high contributions (37.1–53.3%) of the five markers of the sex-related QTL to the phenotypic variation, contributions of the growth-related QTLs were quite low (12.4–17.2%). However, this was not unexpected, as independent QTLs do not have high contributions in such a complicated trait.

We analyzed the QTLs associated with three growth traits (total length, body height, and weight) at two different growth stages in the mandarin fish and found that the QTLs for a single growth trait were distributed on different LGs. Four QTLs supported by four markers for total body length during the first growth stage (FL) were detected on four LGs (LG4, LG10, LG16 and LG19). Investigations in some other fish species also showed that one trait was associated with multiple QTLs. For example, QTLs for the body weight of rainbow trout were detected in seven different LGs [33]. Eleven QTLs for body length in the second (9-months-old) growth stage (L2) of the turbot were detected on eight different LGs [11], and significant QTLs for body weight were detected on five LGs in Asian seabass [34]. Taken together, these results indicate that quantitative variations in the growth traits of fish are controlled by multiple segregated major QTLs.

The QTLs existing in a single LG were found to be associated with different growth traits of the mandarin fish. The QTLs simultaneously controlling the first stage of growth (FL, FW, and FH) were present on LG10, and the QTLs for IL and IH co-existed on LG16. QTLs for growth traits on LG10 and LG16 were distributed in clusters, suggesting a pleiotropic effect or gene cluster in LG10 and LG16 of the mandarin fish.

In this study, a single QTL in some chromosomal regions was found to be associated with two or more growth traits. The markers R1_32378 on LG10 were both significantly correlated with FL, FW, and FH. R1_63885 on LG16 was associated with both IL and IH. Similar phenomena have been observed in some other types of fish. For example, in the Asian seabass, Lca371 on LG2 was shown to be associated with body weight, total length, and standard length [35]. Additionally, in the turbot, the SNP locus SM_343 on LG1 displayed a significant QTL effect during the first and second growth periods and was responsible for two or more traits with an LOD of greater than 5.0 [10]. SNP0626 on LG19 was significantly related to both body weight and body length in the common carp [36]. These findings demonstrate that some single QTLs were related to multiple traits, suggesting that the genes related to such QTLs may display pleiotropic effects, controlling a series of related traits.

For the first growth stage and the increased values of the two stages of the mandarin fish, 6 and 5 QTLs related to growth traits were detected, respectively (Table 3). However, no QTLs were found to be the same. A similar phenomenon was also observed in the turbot. There were 11 QTLs that were significantly related to the body length during the second (9-months-old) growth stage and 21 QTLs significantly related to the body length in the third (12-months-old) growth stage; however, these QTLs were absolutely different and were distributed on different LGs [10]. These data suggest that fish growth is controlled by different genes at different stages.

## Conclusion

In this study, we constructed a high-density genetic linkage map with 3283 SNPs using the ddRAD-Seq technique. The interlocus distance in the map was 0.61 cM. Using this map, one QTL for sex determination, supported by five markers, was detected on LG23, and 11 putative QTLs for growth were found on four LGs, suggesting the involvement of a single LG in the sex-determination genes and multiple genes affecting the growth traits. Although we were unable to map the sex-determination genes or the growth-controlling genes because of the unavailability of the complete genomic DNA sequence of the mandarin fish, the present study provides useful genomic resources for molecular dissection of complex traits and will facilitate de novo genome sequence assembly and genetic improvement of the mandarin fish.

## Methods

### Mapping population

A full-sib family of the mandarin fish was established and used for the development of a genetic linkage map. The male parent was selected from a group of fish derived from a wild population from Hunan province, and the female parent was chosen from a cultured population from YuShun fish farm. Fish breeding and cultivation were performed at YuShun Fisheries Company in Qinxin County, Guangdong Province, China.

Artificial fertilization was performed by mixing the eggs and sperm from the mature broodstock immediately for about 10 s. The fertilized eggs were incubated and nurtured in a circular hatching channel for 60 days. Thereafter, 2000 fries were marked with passive integrated transponder (PIT) tags, transferred to an earth pond, and cultivated for 135 days. The fish were fed thrice daily at around 7:00 a.m., 2:00 p.m., and 6:00 p.m. The data for growth-related traits (body weight, total body length, and body height) of the F1 individuals were collected twice. The first growth data were recorded 60 days after hatching, whereas the second growth data were measured at 195 days, at which time the sexes of the F1 individuals were identified.

A small portion of fin tissue from the parents and randomly sampled 230 individuals of F1 offspring was sheared under 2-phenoxyethanol anesthesia. The genomic DNA from the two parents and their offspring was isolated using traditional phenol-chloroform extraction in combination with RNase treatment and was stored at −20 °C [37]. Before the construction of ddRAD libraries, all the DNA samples were quantified using a NanoDrop instrument (Thermo Scientific, DE, USA) and by agarose gel electrophoresis. The genomic DNA from 155 individuals with high purity (OD_260_/_280_ = 1.8 ~ 2.0; OD_260_/_230_ = 1.8 ~ 2.0) and good integrity (molecular size of the primary band >20 kb) was used for the construction of ddRAD libraries. The concentrations of DNA were adjusted to 50 ng/mL using Tris-EDTA buffer.

### Construction and sequencing of ddRAD libraries

The ddRAD libraries were constructed according to the method described by Peterson et al. (2012) [24]. Briefly, 500 ng of DNA template from each individual was double-digested using the restriction enzymes *Eco*RI and *Nla*III (New England Biolabs, Ipswich, MA, USA; 20 U/reaction) in a combined reaction for 30 min at 37 °C. Subsequently, each fragmented sample was purified using a Qiagen MinElute Reaction Cleanup Kit (Qiagen, Valencia, CA, USA) and eluted in 20 μL elution buffer (EB). The fragments were then ligated to P1 (including a unique 4–8-bp multiplex identifier [MID] used to distinguish each individual) and P2 adapters that bound to the *Eco*RI and *Nla*III overhangs, respectively. In each 40-μL reaction, 500 ng DNA, 1 μL P1 adapter (10 mM), 1 μL P2 adapter (10 mM), 1 μL T4 ligase (1000 U/mL), 4 μL of 10X T4 ligation buffer, and double-distilled water were mixed. The ligation was performed in a polymerase chain reaction (PCR) machine using the following conditions: 37 °C for 30 min, 65 °C for 10 min, followed by a decrease in temperature to 20 °C at a rate of 1.3 °C/min. The samples were pooled and size-selected (400–600 bp) from an agarose gel. The DNA product was subsequently purified using a Qiagen MinElute Gel Purification Kit and eluted in 10 μL EB. The paired-end (150 bp) sequencing of the ddRAD products from the 157 individuals was performed using an Illumina HiSeqXten sequencing platform (Illumina, Inc., San Diego, CA, USA). The sequencing data for each individual were extracted according to the specific MID.

### SNP discovery and genotyping

We first filtered out Illumina short reads lacking sample-specific MIDs and the expected restriction enzyme motifs. Thereafter, the reads were filtered on the basis of their quality score using *Trimmomatic* (v0.32) [38] in three steps: (1) removal of adapters; (2) removal of reads with bases from the start or end of a read, if below the quality threshold 3; and (3) scanning of the reads with a 4-bp sliding window, removing the read when the average Phred quality per base was below 15.

The *STACKS* (Version 1.32) pipeline [39] was used to assemble the loci, de novo, from the sequencing data for SNP calling. *USTACKS*, *CSTACKS*, *SSTACKS*, and *GENOTYPE* programs were used to create libraries of loci, i.e., one for each individual and one for all the loci shared among the individuals. Minimum depth of coverage required to create a stack is 3; Maximum distance (in nucleotides) allowed between stacks is 3; 2 mismatches allowed between sample loci when build the catalog. The detailed parameters used were as follows (Additional file 8: Table S7):

USTACKS: -t gzfastq -i -m 3 -M 3 -p 15 -d -r –f –o

CSTACKS: -b 1 –o –s –n 2 –p 15

SSTACKS: -b 1 –c –p 15

GENOTYPE: -b 1 –P -r 1 -c -s -t CP

Only the miss rates (Number of samples with none genotype information/Number of total samples) less than 10% and biallelic SNPs were selected to avoid sequencing errors using custom *PERL* scripts (https://github.com/Niuyongchao/Fish_linkage_map).

### Linkage map construction

A linkage map was constructed using JoinMap 4.1 [40]. The linkage group assignments were made under the logarithm of odds (LOD) score limit of 6.0. The regression mapping algorithm and Kosambi’s mapping function were used for map construction with the following settings: Rec = 0.4, LOD = 1.0, Jump = 5. The resulting linkage maps were drawn using a custom Perl script (https://github.com/Niuyongchao/Fish_linkage_map).

### QTL mapping

The QTLs were identified using MapQTL 5.0 [41] with multiple QTL mapping (MQM). Automatic cofactor selection (backward elimination, *P* < 0.05) was used for the detection of significantly associated markers as cofactors. The LOD significance threshold levels were determined by Permutation Test on the basis of 1000 permutations at significance level of *P* < 0.05. The location of each QTL was determined according to its LOD peak location and surrounding region. The percentage of the phenotypic variance explained by a QTL (R2) was estimated at the highest probability peak. The QTL results were drawn using a custom Perl script (https://github.com/Niuyongchao/Fish_linkage_map).

## Additional files

Additional file 1: Table S1. Data production of ddRAD sequencing for each individual. (XLS 33 kb)

Additional file 2: Table S2. Polymorphsim SNP markers and their association sequence information. (XLSX 2085 kb)

Additional file 3: Table S3. Tabular representation of the integrated map of Madarin fish. (XLS 276 kb)

Additional file 4: Table S4. Tabular representation of the maternal map of Madarin fish. (XLS 140 kb)

Additional file 5: Figure S1. Linkage group lengths and marker distributions of the maternal map of mandarin fish. **Figure S2.** Linkage group lengths and marker distributions of the paternal map of mandarin fish. **Figure S3.** The QTL curve trends of growth-related traits of the mandarin fish. (PDF 258 kb)

Additional file 6: Table S5. Tabular representation of the paternal map of Madarin fish. (XLS 192 kb)

Additional file 7: Table S6. KS Test with SPSS software. (XLS 45 kb)

Additional file 8: Table S7. The detailed parameters of the STACKS (Version 1.32). (XLSX 9 kb)

## Abbreviations

ddRAD-Sequencing: double digest restriction site associated DNA sequencing
QTL: Quantitative trait locus
SNP: Single nucleotide polymorphism
LOD: Logarithm of odds
