# Supplementary material for: Construction of a high-density linkage map and mapping of sex determination and growth-related loci in the mandarin fish (Siniperca chuatsi)
Source: BMC Genomics. 2017 Jun 6;18:446. doi: 10.1186/s12864-017-3830-3 (PMC5461734; doi:10.1186/s12864-017-3830-3)
Supplement: Supplementary file 5 — Linkage group lengths and marker distributions of the maternal map of mandarin fish. Figure S2. Linkage group lengths and marker distributions of the paternal map of mandarin fish. Figure S3. The QTL curve trends of growth-related traits of the mandarin fish. (PDF 258 kb) [file 12864_2017_3830_MOESM5_ESM.pdf]

## Supplemental Figure Legends

**Supplemental Figures 1. Linkage group lengths and marker distributions of the maternal map of mandarin fish.**

**Supplemental Figures 2. Linkage group lengths and marker distributions of the paternal map of mandarin fish.**

**Supplementary Figure 3. The QTL curve trends of growth-related traits of the mandarin fish.**

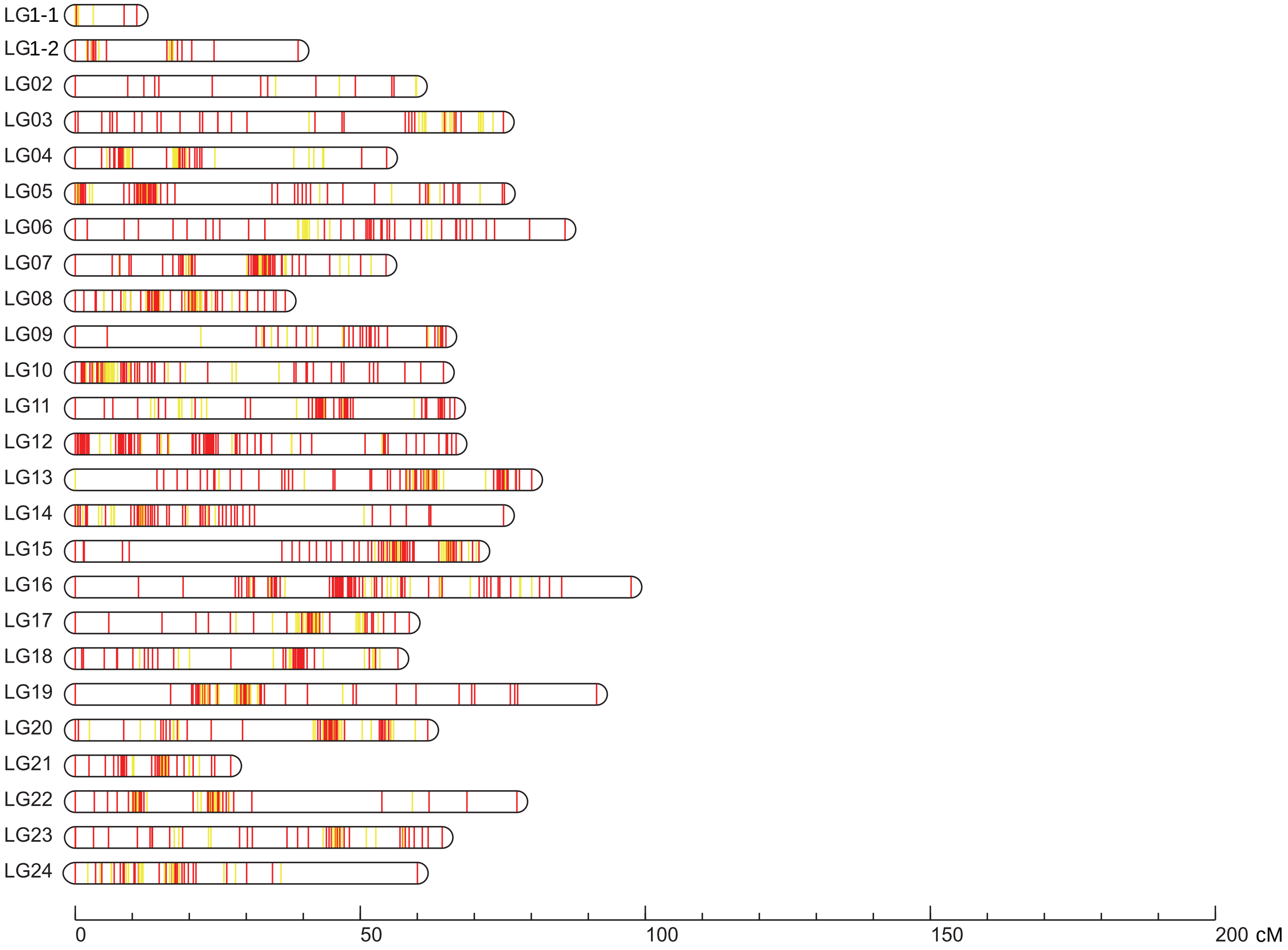

LG1-1
LG1-2
LG02
LG03
LG04
LG05
LG06
LG07
LG08
LG09
LG10
LG11
LG12
LG13
LG14
LG15
LG16
LG17
LG18
LG19
LG20
LG21
LG22
LG23
LG24
0
50
100
150
200 cM

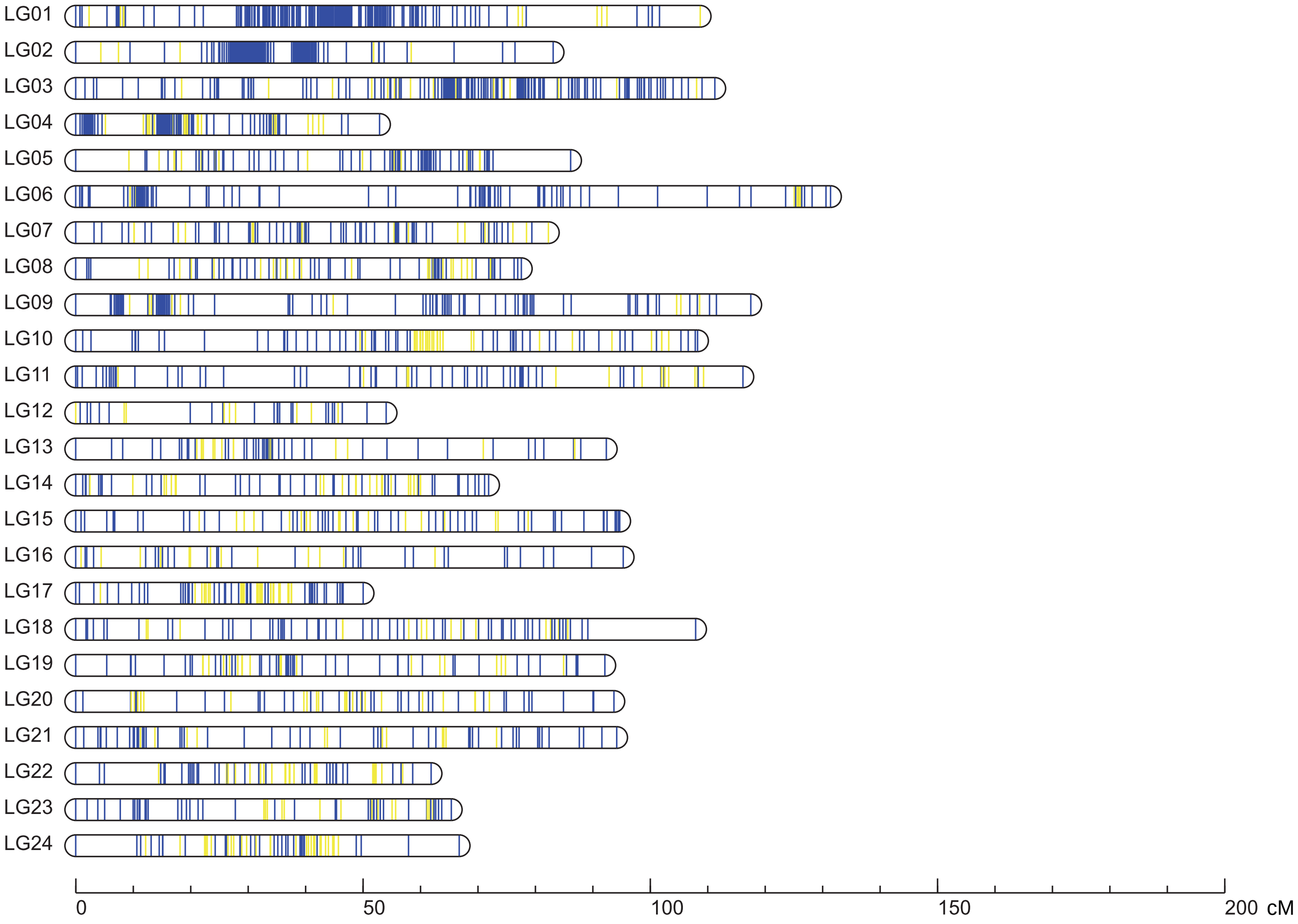
LG01
LG02
LG03
LG04
LG05
LG06
LG07
LG08
LG09
LG10
LG11
LG12
LG13
LG14
LG15
LG16
LG17
LG18
LG19
LG20
LG21
LG22
LG23
LG24
0
50
100
150
200 cM

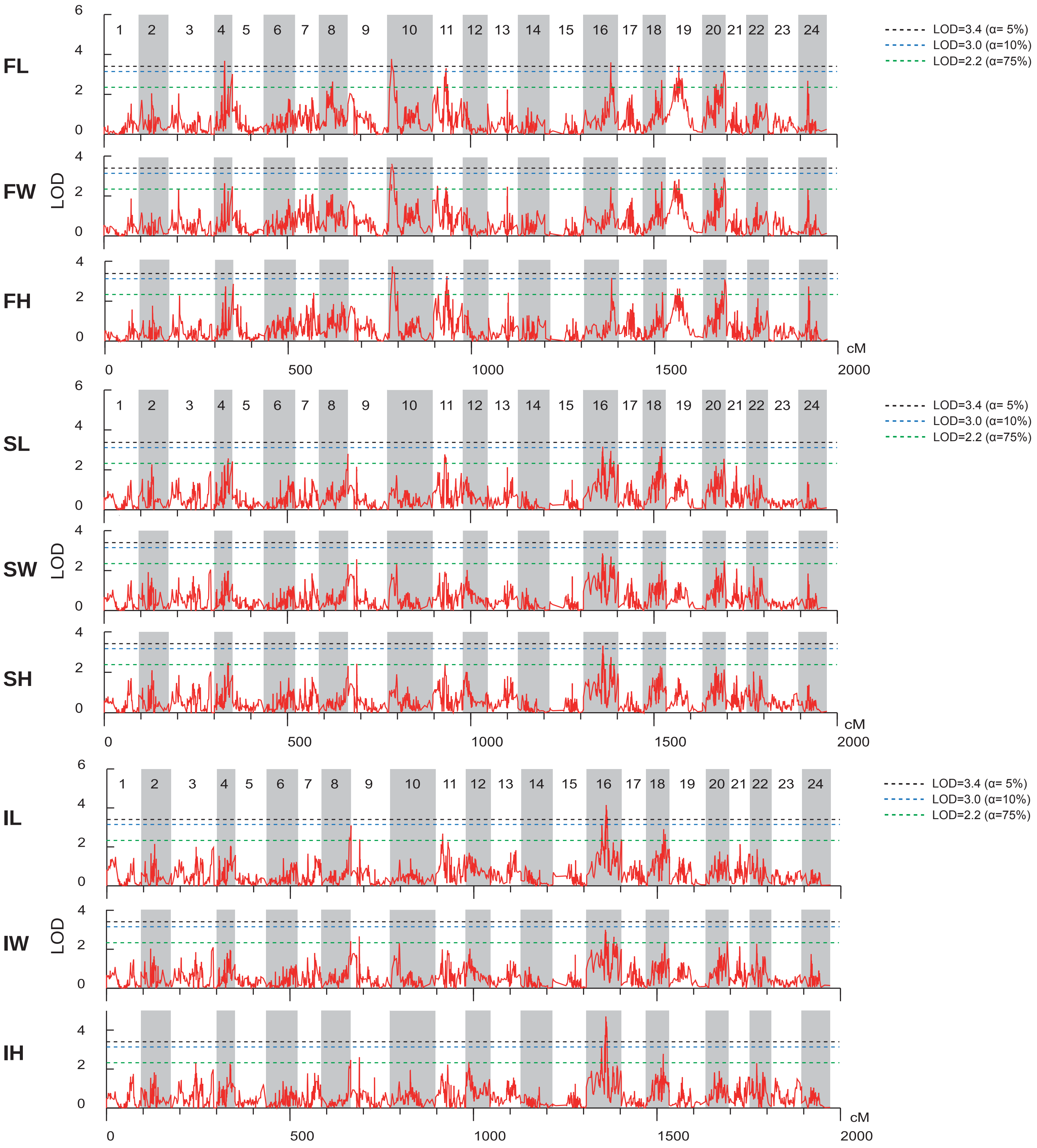

FL
FW
FH
SL
SW
SH
IL
IW
IH
LOD
cM
0
500
1000
1500
2000
1 2 3 4 5 6 7 8 9 10 11 12 13 14 15 16 17 18 19 20 21 22 23 24
LOD=3.4 (α= 5%)
LOD=3.0 (α=10%)
LOD=2.2 (α=75%)
